# Self-reported hypertension as a predictor of chronic health conditions among older adults in Ghana: analysis of the WHO Study on global Ageing and adult health (SAGE) Wave 2

**DOI:** 10.11604/pamj.2020.36.4.21489

**Published:** 2020-05-04

**Authors:** John Tetteh, Kow Entsua-Mensah, Alfred Doku, Sheriff Mohammed, Swithin Mustapha Swaray, Martin Amogre Ayanore, Alfred Edwin Yawson

**Affiliations:** 1 Department of Community Health, University of Ghana Medical School, College of Health Sciences, Korle-Bu, P.O. Box 4236, Accra, Ghana; 2 National Cardiothoracic Centre, Korle-Bu Teaching Hospital, Accra, Ghana; 3 Department of Surgery, University of Ghana Medical School, College of Health Sciences, Korle-Bu, Ghana; 4 Department of Health Policy Planning and Management, School of Public Health, University of Health and Allied Sciences, Ho, Ghana

**Keywords:** Older adults, self-reported hypertension, chronic conditions

## Abstract

**Introduction:**

Hypertension has been identified as a significant predictor of many chronic health conditions. Body Mass Index (BMI) and Quality of Life (QoL) are key determinants of hypertension especially among elderly populations. In this study, we examined the effect of self-reported hypertension (SRH) on chronic health conditions and quality of life among older adults in Ghana.

**Methods:**

The WHO Study on Global Ageing and Adult Health Wave 2 data for Ghana, collected from 2014 to 2015 was applied in this study. Data for older adults aged 50 years and above were analyzed. Weighted descriptive and inferential analyses were performed using Stata 14. We predicted any potential associations between SRH and chronic health conditions using a corrected chi-square and Coarsened Exact Matching with adjusted odds ratios.

**Results:**

The prevalence of SRH among older adults in Ghana was 15.8%. This was significantly associated with sex, marital status, religion, place of residence, working status, location/region, health status BMI, and QoL. In all, older adults with poor health status, obese state and high QoL had 3.15, 2.17 and 2.76 odds of SRH respectively [AOR(95%CI)p-value=3.15(1.65-6.02)0.001, 2.17(1.31-3.59)0.003 and 2.76(1.04-7.31)0.041)]. In addition, older adults with SRH were at increased risk of reporting chronic conditions such as stroke, angina, diabetes and cataract.

**Conclusion:**

Overall, a key observation from this analysis is that SRH (and not only clinically diagnosed hypertension) is significantly associated with co-morbidities. In Ghana, older adults with SRH have increased risk of co-morbidities including diabetes, stroke, angina, and cataract. Interventions to improve the awareness and early detection of hypertension at the population level is key. Controlling hypertension at the population level will reduce prevalence of chronic conditions and increased protection.

## Introduction

Hypertension is a major risk factor for cardiovascular diseases worldwide [1]. The 2017 Global Burden of Disease (GBD) indicated that hypertension was an important public-health challenge worldwide and was estimated to affect 1.56 billion individuals by 2025 with an increased global prevalence of 60% [1, 2]. Hypertension is a chronic multifactorial disease whose detection is often delayed due to its slow and silent progression [2]. However, this asymptomatic chronic disease in most people requires optimal control usually through disciplined adherence to prescribed medications. Without optimal control, there is increased risk of developing cardiovascular, cerebrovascular and renal complications [3].

In sub-Saharan Africa (SSA), the prevalence of hypertension and pre-hypertension is high, with an estimated prevalence rate of 25.9%. This, however, differs by population group [4]. The 2014 Ghana Demographic Health Survey (GDHS) reported a hypertension prevalence rate of 13% among adults [5] in the reproductive age, while the WHO Study on Global AGEing and Adult Health (SAGE), Wave 1 in Ghana reported SRH prevalence rate of 14.2% among older adults 50 years and above [6]. Age is universally associated with hypertension, and in Africa, the greatest burden of hypertension is borne by persons 50 years and above [7]. Increasing age, is a major cause of reduction in the quality of life (QoL) among older adults. [8]. The QoL which is an individual´s perception of happiness and satisfaction with life as well as position in life and value systems in relation to expectations, values and concerns with physical health may be severely compromised [9]. Low QoL affects the psychological well being and socio-demographic functions [10]. Invariably, QoL is an important predictor of treatment outcomes among older adults with hypertension and QoL among older adults with hypertension has been shown to be worse than those without hypertension [10, 11].

However, hypertension among older adults may not be detected early or may not be adequately managed [12]. The consequences of this on the health and social well being of these older persons may be dire. Imperatively, measures needed to be taken for early diagnosis of hypertension and to prevent its complications is essential at the population level, considering that the prevalence of hypertension is estimated to increase [2]. Globally, there is substantial connection between hypertension and other chronic health conditions which reflect in their etiology and disease mechanisms. Hypertension is common among patients with diabetes [13] and stroke [14] . It has been observed in previous studies that 51% of stroke deaths are attributable to hypertension [15]. Hypertension is associated with other chronic conditions that affect the QoL of older persons including eye conditions such as cataract. This impairs the daily functioning of the older adults and affects their quality of life [16]. This analysis determined the prevalence of SRH, predictors of hypertension and quantified the effect of SRH on stroke, angina, diabetes and cataract among older adults in Ghana.

## Methods

**Study setting:** the study was conducted in Ghana with an estimated population of 29,694,793 as at the year 2018 [17, 18] and 5,865 established health facilities across the country [19].

**Data and study participants:** WHO Study on Global AGEing and Adult Health (SAGE) Wave 2 for Ghana dataset was used in this study. SAGE is a longitudinal data on the health and well-being of adult populations as well as the ageing process conducted by WHO. Between 2014 and 2015, SAGE Wave 2 country studies were conducted in six countries; China, Ghana, India, Mexico, Russian Federation and South Africa [20]. Two target populations were measured in SAGE Wave 2. A large sample of persons aged 50 years and older (focus group for SAGE) and a smaller comparative sample of persons in reproductive age group (aged 18-49 years). Households were classified into mutually exclusive categories where one or more persons aged 50 years and older were selected from these households and classified as 50+ household. The presence of one person aged 18-49 years in a household classified as an 18-49 household. In the older households, all persons aged 50 years and older were invited to participate whist proxy respondents were identified for respondents who were unable to respond for themselves. Multistage cluster sampling was used for Ghana Wave 2 with 250 Primary Sample Units and 20 strata [6, 20]. Detailed study design and procedure for data collection adopted in SAGE wave 2 is well described by Kowal *et al.* (2012) [21]. A total of 4,735 persons, comprising older adults (50+ years) and persons in their reproductive age (18-49 years) were interviewed. In line with the aim of the study, data for persons below 50 years and missing responses for SRH were excluded. A final sample of 2,335 adults aged 50 years and above were included in this study.

**Dependent variables:** self-reported hypertension (SRH) was considered the primary outcome variable. Co-morbidities including stroke, angina, diabetes and cataract were also considered as outcome variables. The SRH was defined ‘as ever been told by a doctor or health care professional that you have high blood pressure (hypertension)´.

**Independent variable:** from the original dataset, sex, marital status, religion, educational attainment, residence, region of residence and employment status were extracted for the sociodemographic variables. BMI was estimated from a measured weight (kg) and height (metres) and categorized into <18.5, 18.5-24.9, 25-29.9 and 30 and above as underweight, normal, overweight and obesity respectively. Perceptions on QoL and well-being using the 8-item WHO Quality of Life measure (WHOQoL) from standard questions were used to grade level of life satisfaction. The 8-standard questions were recoded as satisfied=1 and not satisfied=0. An index variable was created for the recoded 8-standard questions with scores ranging from 0-8. These scores (continuous variables) were further reclassified into 0, 1-5, 6-7 and 8 as poor satisfaction, low satisfaction, moderate satisfaction and very satisfied respectively.

**Data analysis:** descriptive statistics involved a weighted two-way observational row percentage table involving independent variables associated with SRH. This was done with a corrected chi-square value taking into consideration the design nature of SAGE Wave 2. The second step of analysis involved inferential statistics involving logistics regression to assess risk factors that are associated with SRH. Coarsened Exact Matching (CEM) method of analysis was carried to improve and reduce imbalances in estimating causal effects. This was conducted to control for some or all of the potentially confounding influence of pretreatment control variables by reducing imbalances between the treated and control groups. This Monotonic Imbalance Bounding (MIB) matching method which balances between the treated (SRH) and control groups (non-SRH), was employed to adjustthe influence of the imbalance of one variable on the maximum imbalance of any other [22]. In this analysis, SRH predictors identified included; Age, Place of residence, Region, Health status, BMI and QoL. These were modeled to reduce the imbalances between them (Table 1).

**Table 1:** CEM weighted balance report before and after matching

Matching variable	L1	mean	L1	mean
Age	0.07	0.03	8.60E-16	5.30E-15
Place of residence	0.30	-0.30	8.60E-16	4.40E-16
Region	0.24	-0.20	6.70E-16	1.10E-14
Health status	0.09	0.24	4.30E-16	2.20E-15
BMI	0.28	0.49	1.10E-15	6.20E-15
QoL	0.05	-0.01	5.00E-16	6.20E-15
**Overall imbalance**	**0.76**		**5.63E-16**	

**NOTE**: Total match sample among the treatment (SRH) =219. Total match sample among controls (non-SRH)=542. E=exponent

**Figure 1:**
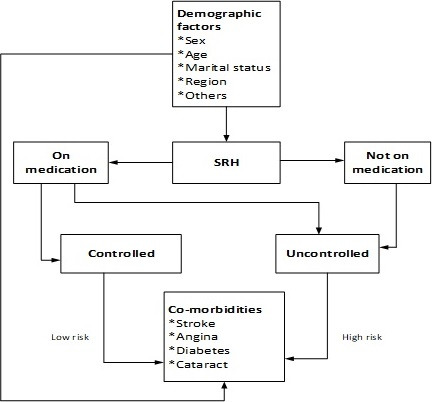
Framework defining the impact of self-reported hypertension on chronic conditions among older adults

Overall 76% of imbalance existed among the independent variables before matching. However, after CEM matching, imbalances reduced to almost 0% (5.63E-16) (Table 1). After preprocessing the data with CEM, sensitivity analysis was applied involving Logistics regression, weighted with CEM estimations to estimate the risk of SRH on stroke, diabetes, angina and cataract adjusting for non-predictors of SRH which includes; sex, marital status, religion and working status. The concept and process involved in conducting this analysis is demonstrated in Figure 1; that older adults with SRH can either be controlled with medication or uncontrolled without medication. The risk of stroke, angina, diabetes and cataract can be either low or high among older adults with SRH in relation to management of the condition, whilst the demographic predictors can also directly affect the health condition.

**Ethical requirements:** the SAGE study was approved by the World Health Organization's Ethical Review Board (reference number RPC149) and the Ethical and Protocol Review Committee, College of Health Sciences, University of Ghana, Accra, Ghana. Written informed consent was obtained from all study respondents.

## Results

Data on a total of 2,335 older adults 50 years and above were analyzed (Table 1). The study found a prevalence of 15.8% for SRH among the older adults in Ghana. Sex differential in SRH existed and was relatively higher among females (20.3% vs. 11.6%). Those in the age group 60-69 years had the highest prevalence of SRH (18.5%). Older adults who reported marital status as separated, had the highest prevalence of SRH (22.5%). In addition, rural-urban disparities existed in the prevalence of SRH and was relatively higher among urban residents (23.1% vs. 8.1%), while unemployed older adults had relatively higher SRH, than those who were working (19.8% vs. 13.2%) In terms of geographical location, older adults living in the Volta region had the highest risk of SRH (51%), compared with those living in the other nine administrative regions in Ghana. Unsurprisingly, poor perception of health status was associated with a higher SRH among the older adults (28.7%) and SRH was highest among obese individuals (34.2%) as compared with other BMI categories (Table 2). Older adults belonging to no identifiable religion had the highest prevalence of SRH (18.3%)

**Table 2:** Demographic characteristics of older adults with self-reported hypertension in Ghana n (%) Weighted %

Demographic variable	Total	Yes	Design base χ2
	N=2335(100)	N=329(15.8)	
	n(%)	n(%)	
**Sex**			7.97**
Male	1106(100)	106(11.6)	
Female	1229(100)	223(20.3)	
**Age**			1.31
50-59	613(100)	75(13.4)	
60-69	796(100)	121(18.5)	
70-79	609(100)	93(17.1)	
80+	317(100)	40(14.7)	
**Marital status**			4.09*
Never married	75(100)	10(14.7)	
Married	1164(100)	127(13.0)	
Separated	298(100)	57(22.5)	
Widowed	798(100)	135(18.7)	
**Religion**			3.71*
None	73(100)	10(18.3)	
Christian	1716(100)	168(17.6)	
Islam	408(100)	38(9.2)	
Primal indigenous	138(100)	13(9.9)	
**Place of residence**			59.7***
Urban	950(100)	216(23.1)	
Rural	1385(100)	113(8.1)	
**Working status**			5.52*
Yes	1449(100)	158(13.2)	
No	835(100)	155(19.8)	
**Region**			12.92***
Ashanti	393(100)	70(15.8)	
Brong Ahafo	236(100)	22(9.6)	
Central	313(100)	28(9.1)	
Eastern	188(100)	24(10.6)	
GT. Accra	254(100)	89(35.9)	
Northern	215(100)	7(3.8)	
Upper East	118(100)	7(5.8)	
Upper West	91(100)	9(7.0)	
Volta	211(100)	31(51.0)	
Western	316(100)	42(14.4)	
**Health status**			7.04***
Very good	1349(100)	163(13.4)	
Moderate	715(100)	111(15.9)	
Bad	268(100)	55(28.7)	
**BMI**			17.53***
Underweight	293(100)	21(8.6)	
Normal	1232(100)	115(10.8)	
Overweight	428(100)	97(19.8)	
Obesity	2539100)	70(34.2)	

NOTE:

* =p-value ≤0.05

** =p-value ≤0.01 and

*** =p-value ≤0.000

In all, differences in SRH among older adults in Ghana were significantly associated with sex, marital status, religion, place of residence, employment status, geographical location/region of residence, health status and BMI (Table 2). Interestingly, among the 329 older adults who self-reported hypertension, only 94 (27.4%) were on antihypertensive medication(s). Older people residing in rural areas had a 43% chance of developing hypertension compared with those who lived in urban areas [aOR(95%CI)=0.43(0.29-0.62)0.000]. Again, older adults aged 70-79 were 88% more likely to have compared with the relatively younger individuals [aOR(95%CI)=1.88(1.07-3.21)0.027]. The other nine administrative regions had a lesser risk of SRH compared with Greater Accra region (as in Table 3, Table 3 (suite)). Older adults who rated their health status as bad were 3.2 times more likely to have SRH [aOR(95%CI)p-value=3.15(1.65-6.02)0.000] whilst obese older adults were 2.3 times more likely to self-report hypertension [aOR(95%CI)p-value=2.25(1.32-3.85)0.003]. Older adults who were highly satisfied with their life were 3.0 times more likely to self-report hypertension [aOR(95%CI)p-value=3.01(1.09-8.32)0.034] as compared with older adults with poor life satisfaction i.e. the higher the QoL, the higher the risk of SRH (Table 3, Table 3 (suite)).

**Table 3:** Demographic risk factors associated with self-reported hypertension in the sample population

Characteristics	Risk factor predictors	AOR	P-value	95% CI	
				Lower	Upper
**Self-reported hypertension**	**Sex**				
	Male	**Ref**			
	Female	1.26	0.422	0.72	2.21
	Age				
	50-59				
	60-69	1.93	0.046	1.01	3.67
	70-79	1.88	0.027	1.07	3.29
	80+	1.45	0.287	0.73	2.85
	**Marital status**				
	Married	**Ref**			
	Never married	0.95	0.894	0.42	2.12
	Separated	1.35	0.179	0.87	2.09
	Widowed	1.32	0.209	0.86	2.03
	**Religion**				
	Christian	**Ref**			
	None	1.73	0.248	0.68	4.39
	Islam	0.80	0.453	0.44	1.45
	Primal indigenous	1.00	0.996	0.45	2.25
	**Place of residence**				
	Urban	**Ref**			
	Rural	0.43	0.000	0.29	0.62
	**Working status**				
	No	**Ref**			
	Yes	1.08	0.750	0.67	1.73
	**Region**				
	GT. Accra	**Ref**			
	Ashanti	0.32	0.000	0.18	0.57
	Brong Ahafo	0.28	0.002	0.13	0.63
	Central	0.21	0.000	0.10	0.41
	Eastern	0.25	0.000	0.12	0.52
	Northern	0.11	0.000	0.04	0.34
	Upper East	0.30	0.040	0.09	0.95
	Upper West	0.31	0.016	0.12	0.80
	Volta	0.45	0.007	0.25	0.80
	Western	0.45	0.007	0.26	0.80

*QoL=Quality of Life

**Table 3 (suite):** Demographic risk factors associated with self-reported hypertension in the sample population

Characteristics	Risk factor predictors	AOR	P-value	95% CI	
				Lower	Upper
	**Health status**				
	Good	**Ref**			
	Moderate	1.23	0.305	0.83	1.81
	Bad	3.15	0.000	1.69	5.91
	**BMI**				
	Normal	**Ref**			
	Underweight	0.87	0.661	0.48	1.60
	Overweight	1.74	0.004	1.20	2.52
	Obesity	2.25	0.003	1.32	3.85
	*QoL				
	Poor	**Ref**			
	Low	1.99	0.093	0.89	4.44
	Moderate	2.29	0.066	0.94	5.55
	High	3.01	0.034	1.09	8.32

* QoL=Quality of Life

In our analysis the prevalence of the selected chronic conditions among older adults with SRH were stroke (2.0%), angina (2.6%), diabetes (3.8%) and cataract (10.0%). Controlling for significant factors associated with SRH and adjusting for; sex, marital status, religion and working status, self-reported hypertension was a significant predictor of stroke, angina, diabetes and cataract among older adults in Ghana (p-value<0.05) as demonstrated in in Table 4. Older adults with SRH had an increased odds for stroke, angina, diabetes and cataract, with significant adjusted odds of 30.9, 4.6, 3.6 and 2.0 respectively compared with those without SRH [aOR(95%CI)=30.85(9.34-102.1), 4.56(1.53-13.55), 3.59(1.46-8.84) and 2.03(1.19-3.46)] (Table 4).

**Table 4:** Effect of self-reported hypertension on selected chronic conditions (stroke, angina, diabetes and cataract) in older adults in Ghana

Explanatory variable	Logistic with CEM			
	Stroke=2.0%	Angina=2.6%	Diabetes=3.8%	Cataract= 10.0%
	aOR[95%CI]	aOR[95%CI]	aOR[95%CI]	aOR[95%CI]
**Hypertension status**				
Not hypertensive	**Ref**	**Ref**	**Ref**	**Ref**
Hypertensive	30.85[9.34-102.1]***	4.56[1.53-13.55]**	3.59[1.46-8.84]**	2.03[1.19-3.46]**
**Sex**				
Male	**Ref**	**Ref**	**Ref**	**Ref**
Female	1.19[0.32-4.48]	1.23[0.51-2.95]	0.62[0.19-1.96]	1.26[0.60-2.63]
**Marital status**				
Married		**Ref**	**Ref**	
Others	0.99[0.28-3.50]	3.55[1.49-8.46]***	1.43[0.44-4.61]	0.68[0.32-1.44]
**Religion**				
Christian	**Ref**	**Ref**	**Ref**	**Ref**
Others	0.07[0.01-0.56]*	0.25[0.03-1.94]	0.29[0.09-0.86]	0.87[0.42-1.83]
**Working status**				
No	**Ref**	**Ref**	**Ref**	**Ref**
Yes	0.60[0.23-1.61]	2.61[0.89-7.65]	0.82[0.36-1.88]	0.48[0.27-0.87]*

* =p-value<0.05,

** =p-value<0.01 and

*** =p-value<0.001

## Discussion

**Prevalence and risk factors associated with self-reported hypertension among older adults:** this analysis found the prevalence of self-reported hypertension (i.e. hypertensives who are aware of their condition) to be 15.8% among older adults in Ghana. This is relatively high as compared with what was reported in WHO SAGE Wave 1 (2008-2009), which reported a prevalence rate of 14.0% [6]. Hypertension is of major public health concern [23-26] and a major risk factor for cardiovascular diseases among adults [23]. A study conducted in Ghana among adults aged 25 years and above identified a 28.3% prevalence rate for hypertension [26] whereas a systematic review identified a range of 19% to 48% prevalence rate of hypertension across all age groups in Ghana [23]. The Ghana Demographic and Health (GDHS) survey of 2014 (coterminous with data collection for WHO SAGE Wave 2) reported a prevalence rate of 13% among men and women in the reproductive age bracket of 15-49 years [5]. This is slightly less than what SAGE wave 2 found, probably due to the relatively younger population used by the GDHS. The identified prevalence of 15.8% is well within that identified in the United States of 13%-19% but lesser than the 24.1% reported by Malta *et al.* among the older adults population of Brazil [2, 27].

Sex differences were identified to have a statistically significant association with SRH, with a female preponderance (20.3% vs. 11.6%). . . Sex differences in the reported rates of hypertension are well documented, males have a higher prevalence for hypertension compared with females of the same age until the 6^th^ decade when females begin to report more [28-30]. Sex differences in hypertension is postulated to be a result of the role of the immune system in mediating sex differences in hypertension where kidneys, the renin-angiotensin system, and developmental programming known to play a role [30,31]. Akin to findings from previous studies, this analysis demonstrated increasing prevalence of SRH with increasing age; adults aged 60 years and above experienced a higher rate SRH (18.5) [24, 26, 28, 32].

In addition, marital status is associated with higher SRH, older adult Ghanaians who were separated from their spouses experienced a higher prevalence rate for hypertension, compared with married older adults. Marriage may afford some protective effect on moderating lifestyle habits such as alcohol intake and poor dietary habits. However, this finding contradicts to an observation that. single individuals are better off in blood pressure profile than unhappy married individuals [33]. Intuitively, emotions and stressors in such a union may increase the risk of hypertension. It has been established that religion improves QoL and lowers risk for developing hypertension [34]. Aligned with this, religion was found to be significantly associated with SRH and that older adults without any form of religious affiliation reported a higher prevalence of SRH (18.3%). Religion, belief systems and health have been demonstrated to be related in all population groups and is statistically correlated to the prevalence of hypertension [35, 36].

Generally, there are inconsistencies in rural-urban disparities in the prevalence of SRH. In our analysis, urban dwellers had relatively higher SRH while rural-dwelling adults were at lower risk and had a decreased predictive probability of SRH. This finding is consistent with the findings of several publications [37-40]. At the same time, it contradicts to findings of other published work where the burden of hypertension among the older adult is significantly higher in rural areas [12, 41-44]. In Ghana, the relative higher prevalence of SRH in urban areas is not surprising. Rural dwellers are more active, are engaged in more physically tasking occupations and have lower prevalence of obesity and inequities in access to health providers [19]. Conversely, urban dwellers may have increased access and exposure to information (traditional and social media) and better awareness of hypertension in the urban areas. This is buttressed by the finding that the most urbanized region, Greater Accra, had the highest prevalence of SRH in Ghana.

In this analysis, adults who were not working reported a greater prevalence of SRH which was similar to a study conducted in Japan where job strain was significantly related to hypertension [45]. This, however, contradicts a retrospective cohort study conducted among 13 European countries where no association was found between working status and hypertension among adults, [46]. Unsurprisingly adults who were within the fifth wealth quintile (richest) had the highest SRH, more likely to be due to increased access, more exposure to information and healthcare. This finding is in keeping with findings by other authors who found that adults from the highest wealth quintile were significantly more likely to have hypertension [47, 48]. Individual’s health status was observed to have a significant association with SRH, such that adults with self-rated bad health conditions were observed to experience a higher SRH and were more likely to be hypertensive. Similarly obese older adults were observed to report high prevalence for hypertension, in keeping with findings from other studies [49-51].

**Effect of Self-reported hypertension on stroke, angina, diabetes and cataract among older adults in Ghana:** this analysis demonstrated clearly that, self-reported hypertension significantly predicts the presence of chronic conditions such as stroke, angina, diabetes and cataract among older adults (in Ghana). We found specifically that self-reported hypertension in older adults increased the risk of stroke, angina, diabetes and cataract compared with older persons without SRH. Other studies [13, 15, 16, 52-57] corroborate this observation. The relationship between hypertension and other chronic condition is well known globally; many people with diabetes have hypertension [13, 52, 53] which increases the risk of stroke [15] and as well as increased risk of cataract [58]. In addition, angina can also be related to hypertension due to the pressure at which the heart has to exert to pump blood [59]. Overall, a key observation from this analysis is that SRH (and not only clinically diagnosed hypertension) is significantly associated with hypertension.

**Limitations:** this analysis used only self-reporting hypertension by older adults. A combination of other methods like consecutive measurement to confirm hypertension could probably have yielded additional information.

## Conclusion

Overall, a key observation from this analysis is that SRH (and not only clinically diagnosed hypertension) is significantly associated with co-morbidities. In Ghana, older adults with SRH have increased risk of co-morbidities including diabetes, stroke, angina, and cataract. Interventions to improve the awareness and early detection of hypertension at the population level is key. Controlling hypertension at the population level will garner reduction in prevalence of chronic conditions and increased protection.

### What is known about this topic

- 14.2% self-reported hypertension exist among older adults 50 years and above;
- The 2014 Ghana Demographic Health Survey reported a hypertension prevalence rate of 13% among adults;
- There is substantial connection between hypertension and other chronic health condition.

### What this study adds

- The prevalence of self-reported hypertension among older adults in Ghana was 15.8%;
- Self-reported hypertension is associated with sex, marital status, religion, place of residence, working status, location/region, health status, BMI, and quality of life;
- Older adults with self-reported hypertension were at increased risk of reporting chronic conditions such as stroke, angina, diabetes and cataract.

## Competing interests

The authors declare no competing interests.
